# Sex differences in metabolic effects of angiotensin-(1-7) treatment in obese mice

**DOI:** 10.1186/s13293-019-0251-9

**Published:** 2019-07-17

**Authors:** Melissa C. White, Amanda J. Miller, Justin Loloi, Sarah S. Bingaman, Biyi Shen, Ming Wang, Yuval Silberman, Sarah H. Lindsey, Amy C. Arnold

**Affiliations:** 1 Department of Comparative Medicine, Penn State College of Medicine, 500 University Drive, Hershey, PA USA; 2 Department of Neural and Behavioral Sciences, Penn State College of Medicine, 500 University Drive Mail Code H109, Hershey, PA 17033 USA; 3 Department of Public Health Sciences, Penn State College of Medicine, 500 University Drive, Hershey, PA USA; 4 Department of Pharmacology, Tulane University, 1430 Tulane Avenue, New Orleans, LA #8683 USA

**Keywords:** Obesity, Angiotensin, Sex, Gender, Insulin, Glucose homeostasis, Drug delivery, Mouse models

## Abstract

**Background:**

Angiotensin-(1-7) is a beneficial hormone of the renin-angiotensin system known to play a positive role in regulation of blood pressure and glucose homeostasis. Previous studies have shown that in high-fat diet (HFD)-induced obese male mice, circulating angiotensin-(1-7) levels are reduced and chronic restoration of this hormone reverses diet-induced insulin resistance; however, this has yet to be examined in female mice. We hypothesized angiotensin-(1-7) would improve insulin sensitivity and glucose tolerance in obese female mice, to a similar extent as previously observed in male mice.

**Methods:**

Five-week-old male and female C57BL/6J mice (8–12/group) were placed on control diet or HFD (16% or 59% kcal from fat, respectively) for 11 weeks. After 8 weeks of diet, mice were implanted with an osmotic pump for 3-week subcutaneous delivery of angiotensin-(1-7) (400 ng/kg/min) or saline vehicle. During the last week of treatment, body mass and composition were measured and intraperitoneal insulin and glucose tolerance tests were performed to assess insulin sensitivity and glucose tolerance, respectively. Mice were euthanized at the end of the study for blood and tissue collection.

**Results:**

HFD increased body mass and adiposity in both sexes. Chronic angiotensin-(1-7) infusion significantly decreased body mass and adiposity and increased lean mass in obese mice of both sexes. While both sexes tended to develop mild hyperglycemia in response to HFD, female mice developed less marked hyperinsulinemia. There was no effect of angiotensin-(1-7) on fasting glucose or insulin levels among diet and sex groups. Male and female mice similarly developed insulin resistance and glucose intolerance in response to HFD feeding. Angiotensin-(1-7) improved insulin sensitivity in both sexes but corrected glucose intolerance only in obese female mice. There were no effects of sex or angiotensin-(1-7) treatment on any of the study outcomes in control diet-fed mice.

**Conclusions:**

This study provides new evidence for sex differences in the impact of chronic angiotensin-(1-7) in obese mice, with females having greater changes in glucose tolerance with treatment. These findings improve understanding of sex differences in renin-angiotensin mechanisms in obesity and illustrate the potential for targeting angiotensin-(1-7) for treatment of this condition.

## Background

Obesity is a global epidemic that greatly increases risk for developing cardiovascular disease and type II diabetes mellitus (T2DM) [1, 2]. Obesity is a state of chronic energy imbalance that is often accompanied by metabolic derangements such as hyperinsulinemia, hyperglycemia, hyperleptinemia, hyperlipidemia, insulin resistance, and glucose intolerance [3]. Accumulating evidence exists for sex differences in the metabolic phenotype of obesity in both animal models and clinical populations [4–6]. While having higher adiposity at any given body mass index compared with men, premenopausal women are protected from obesity-related metabolic and cardiovascular complications as evidenced by lower blood pressure, less adipose tissue distributed to pro-inflammatory visceral depots, smaller and more insulin-sensitive adipocytes, and greater peripheral insulin sensitivity [4–6].

These sex differences in obesity may be, in part, attributed to the renin-angiotensin system (RAS). Most studies to date have focused on the role of angiotensin (Ang) II in obesity. Ang II is a hormone that activates AT_1_ receptors to promote hypertension, insulin resistance, glucose intolerance, and positive-energy balance [7, 8]. More recently, the peptide hormone Ang-(1-7) and additional enzymes have emerged as a counter-regulatory arm of the RAS [9]. Ang-(1-7) is formed from cleavage of Ang II by Ang converting enzyme 2 (ACE2) or cleavage of Ang I by various endopeptidases. Ang-(1-7) activates *mas* receptors to promote positive metabolic effects in male animal models of obesity, T2DM, and cardiometabolic syndrome. More specifically, Ang-(1-7) improves glucose homeostasis by stimulating intracellular insulin signaling pathways, promoting glucose uptake in peripheral tissues, enhancing glucose-stimulated insulin secretion, protecting pancreatic β-cells, and improving insulin sensitivity and glucose tolerance [10–18]. In addition, Ang-(1-7) improves energy balance and lipid metabolism in male rodents [19–21]. Our laboratory recently showed that in high-fat diet (HFD)-induced obese male mice, chronic Ang-(1-7) treatment reverses whole-body insulin resistance by enhancing skeletal muscle glucose uptake [22].

While emerging research is beginning to include sex as an important biological variable, only a handful of studies have examined the sex differences in Ang-(1-7) effects, with a focus on cardiovascular function [23, 24]. The presence of sex-specific differences in metabolic effects of Ang-(1-7) has yet to be considered. This is particularly important given that sex differences in circulating Ang-(1-7) levels are apparent in obese mice and in healthy clinical populations, with females generally having higher levels of this beneficial hormone [25–27]. In this study, we hypothesized that Ang-(1-7) would improve glucose homeostasis in obese female mice, to a similar extent as previously observed in obese male mice.

## Methods

### Approvals

The Institutional Animal Care and Use Committee at the Penn State College of Medicine approved all procedures.

### General study design

Five-week-old male and female C57BL/6J mice (Jackson Laboratory) were used in this study. Macroenvironmental conditions followed the NIH *Guide for the Care and Use of Laboratory Animals* with a 12:12-h light cycle, controlled humidity, and temperature maintained at approximately 23 °C. Male and female mice were weight-matched and divided into four treatment groups (*n* = 8–12 per group for each sex): (1) control diet, saline-treated; (2) control diet, Ang-(1-7)-treated; (3) HFD, saline-treated; and (4) HFD, Ang-(1-7)-treated. Mice were placed on either HFD (Bioserv F3282; 59% kcal from fat, 26% kcal from carbohydrate (~ 40% sucrose) and 15% kcal from casein-based protein) or control diet (Bioserv F4031; 16% kcal from fat, 63% kcal from carbohydrate (~ 42% sucrose), 21% kcal from casein-based protein) for 11 weeks, with food and water provided ad libitum. After 8 weeks on diet, mice were acclimated to individual cages and implanted with osmotic mini-pumps (Alzet model 2004) for chronic 3-week subcutaneous delivery of Ang-(1-7) (400 ng/kg/min; Bachem) or saline vehicle. During the last week of treatment, intraperitoneal insulin and glucose tolerance tests (ipITT and ipGTT, respectively) were performed. On the last day of treatment, body mass and composition were measured and mice were euthanized via cardiac exsanguination under isoflurane anesthesia for collection of blood and adipose tissue. This protocol including route of administration, doses, and time course is consistent with our previous study in a separate cohort of obese male mice showing that Ang-(1-7) infusion improves whole-body insulin sensitivity as measured by hyperinsulinemic-euglycemic clamp methods [22].

### Body composition measurement

Nuclear magnetic resonance imaging was used to measure fat, lean, and fluid masses in conscious mice (Bruker Minispec), with data reported as percentages of total body mass.

### Insulin and glucose tolerance testing

Whole body insulin action was assessed in conscious mice using standardized non-surgical ipITT and ipGTT procedures. For the ipITT, mice were fasted for 4 h and then injected intraperitoneally with insulin (0.75 U/kg of regular U-100 insulin in phosphate buffered saline; Novolin). A tail vein blood sample was taken at baseline and at 15, 30, 60, 90, and 120 min post-insulin injection to measure blood glucose levels with a glucometer (Prodigy AutoCode). An additional blood sample was taken at baseline with a micro-hematocrit capillary tube (FisherBrand) for measurement of plasma insulin concentration. For the ipGTT, mice were fasted overnight and then injected intraperitoneally with 50% dextrose (2 g/kg). Blood glucose was measured at baseline and at 15, 30, 60, 90, and 120 min post-dextrose injection. Plasma insulin concentration was determined at baseline and at 15 and 120 min post-injection. At least 2 days were allowed between ipITT and ipGTT procedures. Given potential differences in baseline fasting glucose among groups, changes in blood glucose during ipITT and ipGTT procedures were normalized to baseline levels and summarized as an area under the curve (AUC) measurement. Plasma insulin was measured using mouse ultrasensitive ELISA (ALPCO).

### Circulating Ang-(1-7) and Ang II concentrations

Ang peptides were measured in subset of mice (5–9 mice/group females and 8–12 mice/group males), with blood samples collected in a peptidase inhibitor cocktail to prevent in vitro metabolism. Plasma was harvested, stored at − 80 °C, and sent to the Biomarker Analytical Core Laboratory at Wake Forest University for radioimmunoassay analysis of Ang II (IBL-America, Minneapolis, MN) and Ang-(1-7) (custom antibody), as previously described [28]. Due to the large number of samples, three separate assays were run for each peptide. The minimum detectable level of the Ang II assay is 2.0 fmol/mL, with 3.3% intra-assay and 4.8% inter-assay variability. The minimum detectable level of the Ang-(1-7) assay is 2.8 fmol/mL, with 8% intra-assay and 20% inter-assay variability.

### Statistical analysis

Data are presented as mean ± SEM for continuous variables. The extreme outliers were evaluated or corrected if they were detected. For each of the outcomes, the main effects of drug, diet, and gender and their pairwise interactions were considered in multiple regressions with the adjusted *P* values obtained based on Wald tests. All hypothesis tests were two-sided with the significance level of 0.05. Data were analyzed using R software version 3.5.2.

## Results

### Body composition

**Fig. 1:**
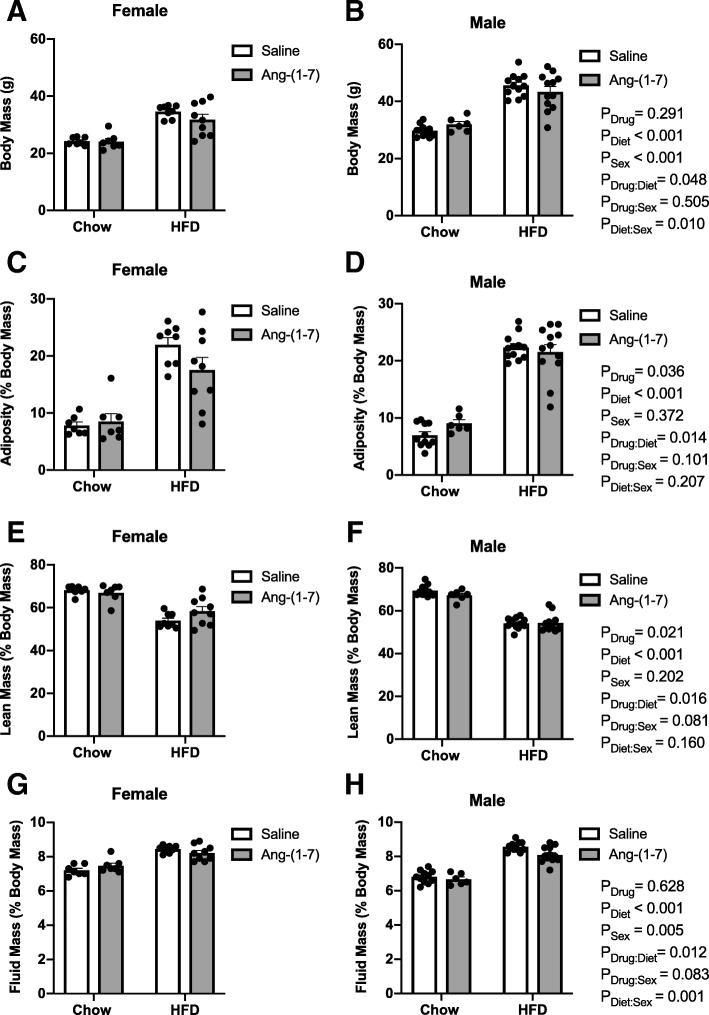
Angiotensin-(1-7) improves body composition in obese male and female mice. Body composition was measured at end of treatment in control diet and high-fat diet (HFD)-induced obese male and female mice chronically treated with angiotensin (Ang)-(1-7) or saline (*n* = 8–12/group). **a**, **b** HFD increased body mass in both sexes; however, males had higher body mass on control diet and HFD compared with females. Ang-(1-7) reduced body mass in obese mice of both sexes. **c**, **d** HFD increased adiposity to a similar extent in male and female mice. Ang-(1-7) reduced adiposity in both obese male and female mice. **e**, **f** HFD reduced lean mass to a similar extent in male and female mice. Ang-(1-7) improved lean mass in obese mice of both sexes. **g**, **h** Female mice had higher fluid mass compared with males, particularly under control diet conditions. HFD increased fluid mass in male and female mice, and chronic Ang-(1-7) treatment reduced fluid mass in obese mice of both sexes. Data are mean ± SE and were analyzed by multiple regression for main effects of sex (*P*_Sex_), diet (*P*_Diet_), and drug (*P*_Drug_) and their pairwise interactions (*P*_Drug:Sex_, *P*_Diet:Sex_, and *P*_Drug:Diet_)

As expected, HFD increased body mass in male and female mice when compared with control diet (Table 1, Fig. 1). Male mice, however, had higher body mass on both control diet and HFD when compared with their female counterparts. The higher body mass in HFD-fed mice of both sexes was due to increases in the percentages of fat and fluid masses and a concomitant decrease in the percentage of lean mass. While there was no impact of sex on adiposity or lean mass, female mice had higher fluid mass compared with males, particularly under control diet conditions. Ang-(1-7) treatment produced small reductions in body mass and adiposity in HFD mice, with no significant main effect of sex or drug to sex interaction. Ang-(1-7) also improved the percentage of lean mass, particularly in HFD mice, with a trend for larger improvements in females. Finally, Ang-(1-7) reduced fluid mass selectively in HFD mice, with no significant sex interaction. In summary, Ang-(1-7) produces small improvements in overall body composition, with no major influence of sex identified for these effects.

**Table 1:** Regression analysis of body composition data in Fig. 1

	Estimate	SE	*T*-statistic	*P* value
Body Mass				
Drug (=Ang-(1-7))	1.643	1.545	1.063	0.291
Diet (=HFD)	15.656	1.465	10.689	0.001
Sex (=female)	− 5.804	1.623	− 3.577	0.001
Drug:Diet	− 3.644	1.810	− 2.013	0.048
Diet:Sex	− 4.861	1.832	− 2.654	0.010
Adiposity				
Drug (=Ang-(1-7)]	3.025	1.417	2.135	0.036
Diet (=HFD)	15.753	1.343	11.729	0.001
Sex (=female)	1.336	1.488	0.898	0.372
Drug:Diet	− 4.183	1.660	− 2.521	0.014
Lean Mass				
Drug (=Ang-(1-7))	− 3.372	1.423	− 2.370	0.021
Diet (=HFD)	− 15.806	1.348	− 11.723	0.001
Sex (=female)	− 1.923	1.494	− 1.288	0.202
Drug:Diet	4.116	1.666	2.471	0.016
Fluid Mass				
Drug (=Ang-(1-7))	− 0.068	0.140	− 0.486	0.628
Diet (=HFD)	1.776	0.133	13.403	0.001
Sex (=female)	0.424	0.147	2.889	0.005
Drug:Diet	− 0.424	0.164	− 2.591	0.012
Diet:Sex	− 0.567	0.166	− 3.418	0.001

Data were analyzed by multiple regressions with the adjusted *P* values obtained based on Wald tests. Results are shown for main effects of drug, diet, and sex and for interactions when reaching statistical significance (*P* < 0.05). *SE* standard error

### Fasting glucose and insulin levels and insulin sensitivity

**Fig. 2:**
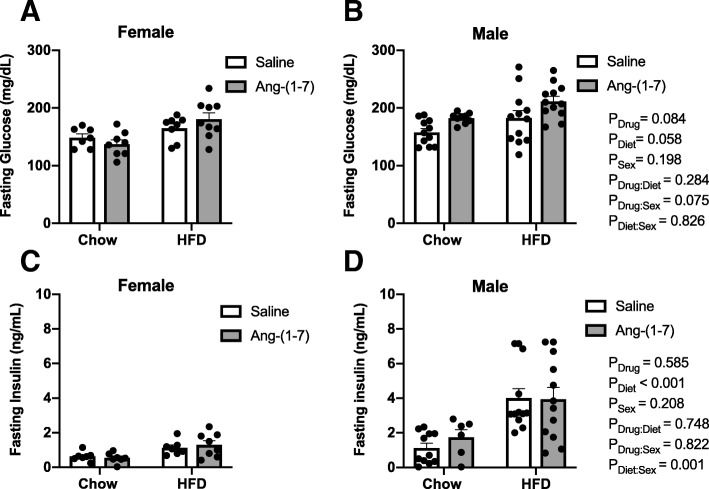
Angiotensin-(1-7) does not alter fasting glucose or insulin levels. Circulating glucose and insulin levels were measured after a 4-h fasting period in control diet and high-fat diet (HFD)-induced obese male and female mice chronically treated with angiotensin (Ang)-(1-7) or saline (*n* = 8–12/group). **a**, **b** HFD tended to produce mild hyperglycemia, which was not different between sexes and not significantly affected by chronic Ang-(1-7) infusion. **c**, **d** HFD produced hyperinsulinemia in both sexes, but to a greater extent in male mice. There was no effect of Ang-(1-7) infusion on insulin levels. Data are mean ± SEM and were analyzed by multiple regression for main effects of sex (*P*_Sex_), diet (*P*_Diet_), and drug (*P*_Drug_) and their pairwise interactions (*P*_Drug:Sex_, *P*_Diet:Sex_, and *P*_Drug:Diet_)

**Fig. 3:**
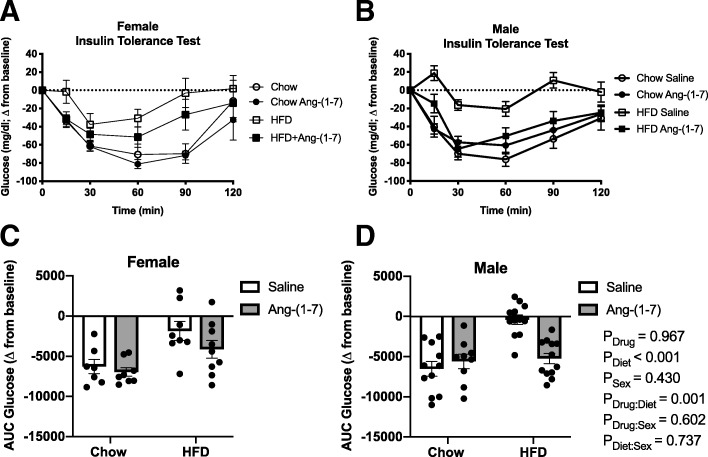
Angiotensin-(1-7) improves insulin sensitivity in obese male and female mice. **a**, **b** Raw data curves showing changes in blood glucose from baseline levels in response to insulin administration over time in control diet and high-fat diet (HFD)-induced obese male and female mice chronically treated with Ang-(1-7) or saline (*n* = 8-12/group). **c**, **d** Data were summarized as an area under the curve (AUC), with a more negative number representing a greater drop in glucose in response to insulin, or increased insulin sensitivity. HFD produced similar insulin resistance in males and females (less negative AUC compared to control diet). While there was no main drug effect among all groups, Ang-(1-7) significantly improved insulin sensitivity in HFD-induced obese male and female mice. Data are mean ± SEM and were analyzed by multiple regression for main effects of sex (*P*_Sex_), diet (*P*_Diet_), and drug (*P*_Drug_) and their pairwise interactions (*P*_Drug:Sex_, *P*_Diet:Sex_, and *P*_Drug:Diet_)

Male and female HFD mice developed a similar mild hyperglycemia, as evidenced by average fasting blood glucose greater than 165 mg/dL, which did not reach statistical significance from control diet-fed mice (Table 2, Fig. 2a, b). There was no significant effect of Ang-(1-7) treatment on glucose levels or interactions with sex or diet. As shown in Fig. 2c, d, HFD increased fasting insulin levels, with no significant main effects of sex or treatment. Obese male mice, however, developed more marked hyperinsulinemia compared with obese female mice. For the ipITT, the decrease in blood glucose levels in response to exogenous insulin administration over the 120-min study period is shown in Fig. 3a, b. A more negative AUC for changes in glucose during ipITT indicates higher insulin sensitivity or a greater drop in blood glucose levels over time in response to insulin. The AUC was less negative in obese male and female mice compared with their lean counterparts suggesting similar levels of insulin resistance in both sexes (Table 2, Figure 3c, d). Ang-(1-7) reversed insulin resistance in HFD-fed mice of both sexes, with no effect on insulin sensitivity in control diet-fed mice.

**Table 2:** Regression analysis of insulin tolerance testing results in Figs. 2 and 3

	Estimate	SE	*T*-statistic	*P* value
Fasting Glucose				
Drug (=Ang-(1-7))	19.421	11.091	1.751	0.084
Diet (=HFD)	20.288	10.511	1.930	0.058
Sex (=female)	− 15.141	11.645	− 1.300	0.198
Fasting Insulin				
Drug (=Ang-(1-7))	0.291	0.530	0.549	0.585
Diet (=HFD)	2.722	0.502	5.422	0.001
Sex (=female)	− 0.707	0.556	− 1.271	0.208
Diet:Sex	− 2.120	0.628	− 3.375	0.001
AUC Glucose				
Drug (=Ang-(1-7))	− 43.09	1045.98	− 0.041	0.967
Diet (=HFD)	5284.48	991.32	5.331	0.001
Sex (=female)	− 872.61	1098.21	− 0.795	0.430
Drug:Diet	− 3998.21	1224.93	− 3.264	0.002

Data were analyzed by multiple regressions with the adjusted *P* values obtained based on Wald tests. Results are shown for main effects of drug, diet, and sex and for interactions when reaching statistical significance (*P* < 0.05). *SE* standard error, *AUC* area under the curve

### Glucose tolerance and endogenous insulin responsiveness

**Fig. 4:**
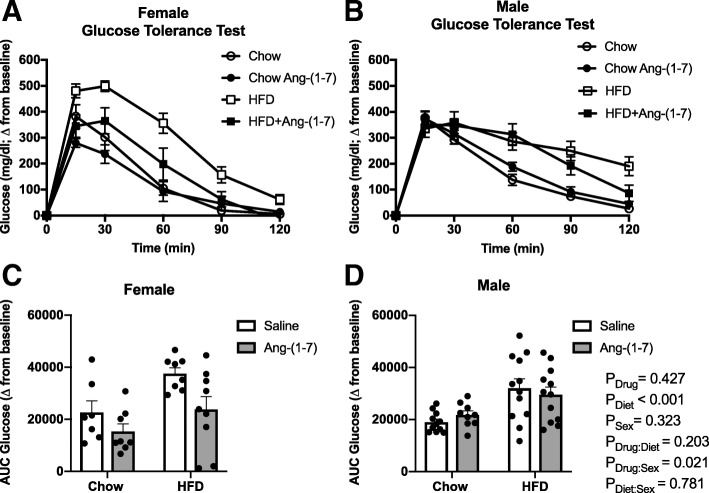
Angiotensin-(1-7) improves glucose tolerance only in obese female mice. **a**, **b** Raw data curves showing changes in blood glucose from baseline levels over time in response to dextrose administration in control diet and high-fat diet (HFD)-induced obese male and female mice chronically treated with Ang-(1-7) or saline (*n* = 8–12/group). **c**, **d** Data were summarized as an area under the curve (AUC), with a more positive number representing higher levels of glucose remaining in the blood over time after dextrose or glucose intolerance. HFD produced similar glucose intolerance in males and females (more positive AUC compared to control diet). Ang-(1-7) selectively improved glucose tolerance in obese female mice. Data are mean ± SEM and were analyzed by multiple regression for main effects of sex (*P*_Sex_), diet (*P*_Diet_), and drug (*P*_Drug_) and their pairwise interactions (*P*_Drug:Sex_, *P*_Diet:Sex_, and *P*_Drug:Diet_)

**Fig. 5:**
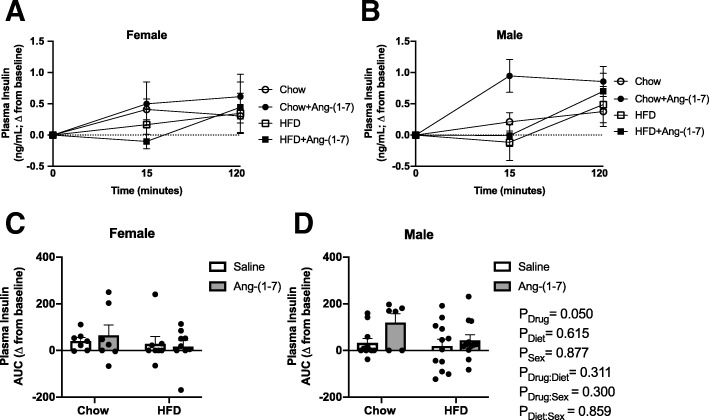
Angiotensin-(1-7) improves glucose-stimulated insulin levels in male and female mice. **a**, **b** Raw data curves showing changes in plasma insulin from baseline levels over time in response to glucose (dextrose) administration in control diet- and high-fat diet (HFD)-fed male and female mice chronically treated with Ang-(1-7) versus saline (*n* = 8–12/group). **c**, **d** Data were summarized as an area under the curve (AUC), with a more positive number representing higher levels of insulin in the blood after glucose administration. There were no differences in increases in insulin levels in response to dextrose between diet and sex groups. Ang-(1-7) increased glucose-stimulated insulin levels, with no interactions with diet or sex. Data are mean ± SEM and were analyzed by multiple regression for main effects of sex (*P*_Sex_), diet (*P*_Diet_), and drug (*P*_Drug_) and their pairwise interactions (*P*_Drug:Sex_, *P*_Diet:Sex_, and *P*_Drug:Diet_)

For the ipGTT, the increase in blood glucose levels in response to exogenous dextrose administration over the 120-min study period is shown in Fig. 4a, b. A more positive AUC value indicates glucose intolerance, meaning blood glucose levels remained increased over time in response to dextrose administration. The AUC was higher in both male and female HFD groups when compared to control diet groups, consistent with glucose intolerance (Table 3, Fig. 4c, d). Ang-(1-7) improved glucose tolerance only in female mice. There was no effect of Ang-(1-7) on glucose tolerance in male or female control diet-fed mice. During the ipGTT, the change in plasma insulin concentration in response to dextrose was also measured, to assess potential changes in glucose-stimulated endogenous insulin secretion (Fig. 5a, b). To account for basal differences among groups, changes in insulin were normalized to baseline levels and summarized as an AUC measurement, with a higher AUC value indicating increased insulin secretion. The AUC for insulin was increased in Ang-(1-7)-infused mice (Table 3, Fig. 5c, d). There were no interactions for Ang-(1-7) effects on insulin levels with diet conditions or sex.

**Table 3:** Regression analysis of glucose tolerance testing results in Figs. 4 and 5

	Estimate	SE	*T*-statistic	*P* value
AUC Glucose				
Drug (=Ang-(1-7))	3075	3849	0.799	0.427
Diet (=HFD)	13239	3648	3.629	0.001
Sex (=female)	4024	4041	0.996	0.323
Drug:Sex	− 10772	4549	− 2.368	0.021
AUC Insulin				
Drug (=Ang-(1-7))	65.692	32.864	1.999	0.049
Diet (=HFD)	− 15.749	31.146	− 0.506	0.615
Sex (=female)	5.348	34.505	0.155	0.877

Data were analyzed by multiple regressions with the adjusted *P* values obtained based on Wald tests. Results are shown for main effects of drug, diet, and sex and for interactions when reaching statistical significance (*P* < 0.05). *SE* standard error, *AUC* area under the curve

### Circulating Ang-(1-7) and Ang II concentrations

**Fig. 6:**
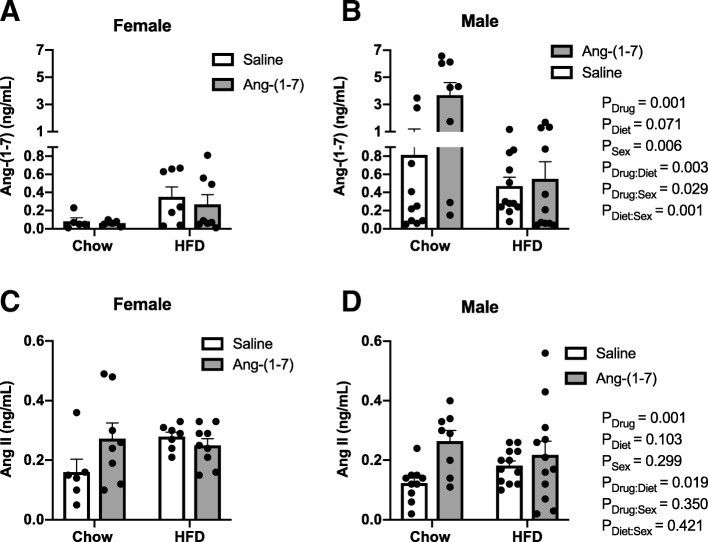
Plasma Ang II and Ang-(1-7) concentrations. Plasma angiotensin (Ang)-(1-7) and Ang II concentrations measured in control diet- and high-fat diet (HFD)-fed male and female mice chronically treated with Ang-(1-7) versus saline (*n* = 7–12/group). **a**, **b** Females exhibited lower circulating Ang-(1-7) concentrations, with no significant effect of diet. As expected, chronic Ang-(1-7) infusion significantly increased plasma levels of this hormone, particularly in chow-fed male mice, as evidenced by interactions of drug infusion with diet and sex. **c**, **d** There were no main effects of diet or sex on plasma Ang II levels. Chronic Ang-(1-7) infusion produced reflexive increases in plasma Ang II levels, with no interactions with diet or sex. Data are mean ± SEM and were analyzed by multiple regression for main effects of sex (*P*_Sex_), diet (*P*_Diet_), and drug (*P*_Drug_) and their pairwise interactions (*P*_Drug:Sex_, *P*_Diet:Sex_, and *P*_Drug:Diet_)

There was a significant main effect for sex for circulating Ang-(1-7) concentrations, with males exhibiting higher levels of the hormone and no diet main effect detected. Similar to our previous study [22], Ang-(1-7)-infused mice had significantly greater circulating Ang-(1-7) compared with saline-treated mice (Table 4, Fig. 6a, b). Significant interactions of Ang-(1-7) infusion with diet and sex were detected, with elevations in this hormone particularly evident in control diet-fed male mice. There were no main effects of diet or sex on circulating Ang II levels, or interactions between diet and sex. Ang-(1-7) infusion elevated endogenous Ang II levels compared to saline-treated mice, with an interaction between drug and diet showing effects most evident in control diet-fed mice (Table 4, Fig. 6c, d).

**Table 4:** Regression analysis of circulating angiotensin peptide results in Fig. 6

	Estimate	SE	*T*-statistic	*P* value
Angiotensin-(1-7)				
Drug (=Ang-(1-7))	2296.6	477.2	4.81	0.001
Diet (=HFD)	− 811.8	441.3	− 1.84	0.071
Sex (=female)	− 1501.1	525.9	− 2.85	0.006
Drug:Diet	− 1770.6	566.1	− 3.13	0.003
Drug:Sex	− 1290.9	576.3	− 2.24	0.029
Diet:Sex	1956.9	584.9	3.346	0.001
Angiotensin II				
Drug (=Ang-(1-7))	148.21	42.61	3.48	0.001
Diet (=HFD)	65.43	39.57	1.65	0.103
Sex (=female)	47.08	45.01	1.05	0.299
Drug:Diet	− 118.79	49.20	− 2.41	0.019

Data were analyzed by multiple regressions with the adjusted *P* values obtained based on Wald tests. Results are shown for main effects of drug, diet, and sex and for interactions when reaching statistical significance (*P* < 0.05). *SE* standard error

## Discussion

The aim of this study was to determine potential sex differences in metabolic effects of chronic Ang-(1-7) treatment in HFD-induced obese mice. The main findings are that (1) male and female mice develop a similar obese metabolic phenotype in response to HFD, with the exception of a milder hyperinsulinemia in females; (2) chronic Ang-(1-7) treatment reduces body mass and adiposity and improves lean mass in obese mice of both sexes, with no effect on body composition in control diet-fed mice; and (3) Ang-(1-7) reverses HFD-induced insulin resistance in both sexes but only improves glucose tolerance in females. These collective data provide new evidence for sexual dimorphism in effects of chronic Ang-(1-7) treatment in obese mice, with females potentially being more responsive in terms of glucose tolerance. These findings advance our limited understanding of sex differences in RAS mechanisms involved in glucose homeostasis and provide new insight for the potential for targeting Ang-(1-7) as a novel therapeutic strategy for metabolic complications in obesity.

The HFD-induced obese mouse has been used extensively as a model for obesity, given its similarity in terms of pathophysiology to the human condition [29]. C57BL/6 mice, in particular, are susceptible to increased adiposity, hyperglycemia, hyperinsulinemia, insulin resistance, and glucose intolerance when chronically exposed to a HFD. Historically, most studies in this model have been performed in males as they develop a more severe degree of obesity and related metabolic complications and to avoid potential estrous-associated physiological alterations [4, 29, 30]. Recent studies, however, have explored sex differences in body composition and glucose homeostasis in this model. For example, one study showed that while HFD-induced obese female mice accumulate more subcutaneous and epididymal fat compared with males, they have reduced circulating insulin levels and develop milder glucose intolerance than their male counterparts [30]. Similarly, HFD-fed female mice are reported to exhibit greater weight gain and adiposity compared with male mice and are protected from obesity hypertension [23]. These findings appear to support clinical literature showing that despite having higher adiposity, females may be protected from obesity-related metabolic and cardiovascular complications.

In the present study, we observed that HFD increases body mass in both sexes but to a greater extent in male mice. Despite lower weight gain, HFD-fed female mice exhibited similar adiposity when compared with males. A limitation of our study is that we did not systematically assess for differences in visceral versus subcutaneous adipose depot distribution between sexes, or in response to diet or drug treatment. Interestingly, we found that female mice develop obesity-induced hyperinsulinemia to a lesser extent compared with male mice, despite having similar mild hyperglycemia. This may suggest obese female mice are more insulin responsive than obese males, as they appear to require less insulin to maintain blood glucose levels; however, we found that HFD produced similar insulin resistance in both sexes when measured by ipITT. The finding that obese female mice were insulin resistant despite lack of marked hyperinsulinemia contrasts what is typically seen in the human population where hyperinsulinemia is an early indicator of prediabetes and T2DM and is closely linked with concurrent insulin resistance [31, 32]. Conversely, genetically altered mice in which insulin secretion is limited are resistant to HFD-induced obesity [33]. Unlike these mice, however, we found that female mice develop obesity and increases in adiposity, suggesting an alternative mechanism of action for their maintenance of normoinsulinemic levels.

Previous studies have shown that Ang-(1-7) reduces body mass and adiposity [13, 20–22] and has protective effects on skeletal muscle composition and function [34], in male rodents. Similar to these findings, we found that Ang-(1-7) improves overall body composition in obese male and female mice by reducing percentage of fat and fluid masses and increasing percentage of lean mass. It is important to note, however, that these mice still remained obese, which may reflect the short 3-week duration of Ang-(1-7) treatment in our study. Since energy balance is tightly regulated, it may take more extended time frames to manifest changes in body mass. In support of this, one study found that male fructose-fed rats supplemented with Ang-(1-7) for 4 weeks had similar weight gain as the corresponding saline group [35]. When the length of treatment was extended to 6 months, however, fructose-receiving rats had similar body mass and adiposity compared with controls. Therefore, extending the length of treatment may result in more profound improvements in body composition in both sexes.

There are conflicting reports involving Ang-(1-7) effects on fasting glucose and insulin levels. One group found that Ang-(1-7) significantly reduces baseline blood glucose, with no effect on basal insulin levels, in male fructose-fed rats [35]. Other studies, however, have shown Ang-(1-7) has no effect on fasting glucose levels with a trend to decrease baseline insulin concentrations [17, 22]. The discrepancy may correlate with differences in species (rats versus mice), obesity models (HFD versus fructose), and length of treatment. Our results showed that Ang-(1-7) has no effect on fasting plasma glucose or insulin levels, regardless of sex or diet received. This is consistent with a recent study from our laboratory showing that a similar duration of Ang-(1-7) treatment did not produce significant effects on fasting glucose or insulin levels, although a trend for a reduction in insulin was observed [22]. The reason for this outcome is unclear but again may reflect Ang-(1-7) therapy duration. Since improvements in insulin sensitivity often occur prior to correction of hyperglycemia, it is possible that longer durations of treatment are needed to manifest changes in glucose and insulin levels. In support of this, a recent study showed changes in plasma insulin at 4 weeks, followed by a reduction in glucose at 9 weeks, after chronic Ang-(1-7) therapy in the db/db diabetic mouse model [36].

Ang-(1-7) improves insulin sensitivity in lean, obese, and diabetic male rodent models via numerous mechanisms including positive effects on intracellular insulin signaling pathways and increasing glucose uptake in peripheral tissues [11–14, 22]. A previous study from our laboratory showed that Ang-(1-7) improves whole-body insulin sensitivity in HFD-induced obese male mice by enhancing glucose uptake within skeletal muscle through increased expression of sarcolemmal glucose 4 transporters (GLUT4) [22]. In the current study, we similarly found that Ang-(1-7) reverses insulin resistance in HFD-induced obese male mice. We expand on these previous findings by demonstrating Ang-(1-7) also improves insulin sensitivity to a similar extent in HFD-induced obese females. The mechanism of action for this return of insulin sensitivity in females is currently unknown but is anticipated to reflect skeletal muscle insulin sensitization similar to what has been previously seen in males [22].

Chronic Ang-(1-7) administration or ACE2 activation also improves glucose tolerance in male rodent models of metabolic syndrome and T2DM [13, 14, 21, 35, 36]. In this study, we found that Ang-(1-7) improved the ability to dispose of exogenous glucose from the bloodstream in HFD-fed female mice, but not in males. Since earlier studies demonstrated that Ang-(1-7) improves pancreatic β cell function to increase glucose-mediated insulin secretion [17, 37, 38], we assessed for insulin receptivity in response to dextrose administration. We found that Ang-(1-7)-treated mice had higher glucose-stimulated insulin concentrations independent of sex or diet. In addition to insulin secretion, glucose tolerance tests induce multiple physiological responses including intestinal glucose absorption, insulin sensitivity, and uptake of glucose in peripheral tissues, glucose effectiveness, and counter-regulatory mechanisms, any of which could account for these sex differences [39]. In addition, while not explored in this study, Ang-(1-7)-mediated vasodilation is more pronounced in women versus men [27], which could serve to increase rate of glucose shuttling to peripheral tissues to enhance glucose tolerance.

There are currently limited studies examining sex differences in circulating Ang peptides in rodent models [23, 24, 40, 41]. In the present study, there were no significant main effects of diet or sex on Ang II concentrations. Similar to our findings, one study showed no difference in Ang II in HFD versus control diet-fed male mice. Another study showed, however, that HFD increases Ang II in males, with no effect on levels of this hormone in females. Similar to our findings, a few studies have shown no sex differences in Ang II levels in normotensive rats and healthy humans; however, others have shown that males have higher levels of Ang II compared with females in obese mice and in control, hypertensive, and diabetic rats. Ang-(1-7) infusion elevated circulating Ang II levels in this study, which was more conspicuous in chow-fed mice and with no sex interaction. Our results parallel previous findings in chow- and HFD-fed groups [22], with this counterintuitive elevation in Ang II perhaps reflecting a physiological balance response.

In terms of Ang-(1-7), a significant diet effect was not detected, although a trend was apparent for HFD to decrease levels in males and increase levels in females. This is consistent with our previous report showing reduced Ang-(1-7) levels in HFD-induced obese male mice. An additional report showed no effect in male mice, but an increase in Ang-(1-7) in female mice in response to HFD as a potential compensatory mechanism to protect against development of hypertension [23]. In this study, we found a significant main effect for sex, with males exhibiting higher levels compared with females, particularly under control diet conditions. This finding is consistent with a previous report in chow-fed mice [23]. It contrasts, however, with studies showing higher circulating Ang-(1-7) concentrations in healthy women and hypertensive rats, and higher renal Ang-(1-7) in female rats [27, 41–43]. Additionally, studies have shown no sex differences in Ang-(1-7) levels in obese mice, normotensive rats, and diabetic rats [23, 40, 41]. Similar to our previous study [22], chronic Ang-(1-7) infusion increased plasma Ang-(1-7) levels, with effects most prominent in males and under chow diet conditions.

Overall, these previous studies have shown inconsistent results for diet and sex effects on circulating Ang II and Ang-(1-7) concentrations. These disparate findings may reflect differences in species (e.g., rats, mice, humans), disease models (e.g., diet-induced obesity, type I diabetes, hypertension, healthy), and assays used (e.g., radioimmunoassay, ELISA). In addition, we observed large variability in Ang peptide levels among individual mice, which may reflect inter-assay variability as well as differences in cohorts.

## Perspectives and significance

In summary, we found that females develop a similar HFD-induced obese phenotype compared with males, with the exception of a milder degree of hyperinsulinemia. Chronic Ang-(1-7) treatment reduced body mass and adiposity and improved lean mass to a similar extent in obese male and female mice. Ang-(1-7) also reversed insulin resistance in both obese male and female mice with no effect on the lean cohort. In contrast to HFD males, however, Ang-(1-7) corrected deviations in glucose tolerance only in the HFD female cohort. This improvement in glucose tolerance with Ang-(1-7) was associated with increased glucose-stimulated insulin secretion when compared to saline-infused mice, which was not dependent on sex. Future studies will examine tissue-specific mechanisms by which Ang-(1-7) improves insulin sensitivity and glucose tolerance in females, the impact of longer durations of treatment, as well as the contribution of sex hormones to these effects. While not assessed in this metabolically focused study, future research should also examine for sex differences in blood pressure responses to chronic Ang-(1-7) treatment in obese mice. These overall findings improve our understanding of sex differences in RAS mechanisms involved in metabolic control in obesity. These findings also provide new insight into the potential for targeting Ang-(1-7) for treatment of obesity and related metabolic complications in an established obese mouse model, with females potentially being more responsive to chronic therapy.

## Data Availability

The datasets used and/or analyzed during the current study are available from the corresponding author on reasonable request.
